# Ceramide-1-phosphate transfer protein (CPTP) regulation by phosphoinositides#

**DOI:** 10.1016/j.jbc.2021.100600

**Published:** 2021-03-26

**Authors:** Yong-Guang Gao, Xiuhong Zhai, Ivan A. Boldyrev, Julian G. Molotkovsky, Dinshaw J. Patel, Lucy Malinina, Rhoderick E. Brown

**Affiliations:** 1 Hormel Institute, University of Minnesota, Austin, Minnesota, USA; 2 Shemyakin-Ovchinnikov Institute of Bioorganic Chemistry, Russian Academy of Sciences, Moscow, Russian Federation; 3 Structural Biology Program, Memorial Sloan-Kettering Cancer Center, New York, New York, USA

**Keywords:** human ceramide-1-phosphate transfer protein (CPTP), *Arabidopsis* accelerated cell death (ACD11) protein, human glycolipid transfer protein (GLTP), GLTP-fold, phosphoinositide regulation, membrane interaction, di-Arg motif, phosphatidylinositol-4-phosphate, phosphatidylinositol-4,5-phosphate, phosphatidylinositol-3-phosphate, ACD11, accelerated cell death-11, AIR, ambiguous interaction restraint, C1P, ceramide-1-phosphate, CPTP, ceramide-1-phosphate transfer protein, FRET, fluorescence energy transfer, GLTP, glycolipid transfer protein, LBD, lipid-binding domain, LTP, lipid transfer protein, OPM, orientation of proteins in membranes, ORF, open reading frame, PH, pleckstrin homology, PS, phosphatidylserine, SL, sphingolipid, SPR, surface plasmon resonance

## Abstract

Ceramide-1-phosphate transfer proteins (CPTPs) are members of the glycolipid transfer protein (GLTP) superfamily that shuttle ceramide-1-phosphate (C1P) between membranes. CPTPs regulate cellular sphingolipid homeostasis in ways that impact programmed cell death and inflammation. CPTP downregulation specifically alters C1P levels in the plasma and *trans*-Golgi membranes, stimulating proinflammatory eicosanoid production and autophagy-dependent inflammasome-mediated cytokine release. However, the mechanisms used by CPTP to target the *trans*-Golgi and plasma membrane are not well understood. Here, we monitored C1P intervesicular transfer using fluorescence energy transfer (FRET) and showed that certain phosphoinositides (phosphatidylinositol 4,5 bisphosphate (PI-(4,5)P_2_) and phosphatidylinositol 4-phosphate (PI-4P)) increased CPTP transfer activity, whereas others (phosphatidylinositol 3-phosphate (PI-3P) and PI) did not. PIPs that stimulated CPTP did not stimulate GLTP, another superfamily member. Short-chain PI-(4,5)P_2,_ which is soluble and does not remain membrane-embedded, failed to activate CPTP. CPTP stimulation by physiologically relevant PI-(4,5)P_2_ levels surpassed that of phosphatidylserine (PS), the only known non-PIP stimulator of CPTP, despite PI-(4,5)P_2_ increasing membrane equilibrium binding affinity less effectively than PS. Functional mapping of mutations that led to altered FRET lipid transfer and assessment of CPTP membrane interaction by surface plasmon resonance indicated that di-arginine motifs located in the α-6 helix and the α3-α4 helix regulatory loop of the membrane-interaction region serve as PI-(4,5)P_2_ headgroup-specific interaction sites. Haddock modeling revealed specific interactions involving the PI-(4,5)P_2_ headgroup that left the acyl chains oriented favorably for membrane embedding. We propose that PI-(4,5)P_2_ interaction sites enhance CPTP activity by serving as preferred membrane targeting/docking sites that favorably orient the protein for function.

Lipid intracellular transport by vesicular and nonvesicular mechanisms helps maintain distinct lipid compositions associated with various cell organelles. Vesicular lipid transport involves budding and fission of vesicles from source membranes followed by trafficking and fusion with destination membranes. Nonvesicular lipid transport occurs *via* lipid transfer proteins (LTPs) that acquire and release their specific lipid cargoes during transient interaction with source and destination membranes (1, 2, 3, 4, 5, 6, 7, 8, 9, 10, 11, 12). Variations in LTP transfer mechanisms include: *i)* shuttling of the amphitropic LTP back and forth through the aqueous milieu between membranes or *ii)* pendulum-like swinging of membrane-associated protein containing an LTP domain between closely apposed membranes. LTPs involved in the nonvesicular trafficking of sphingolipids (SLs) between membranes include ceramide transfer protein (CERT) (13, 14), certain SL activator proteins (15, 16, 17, 18), and members of the glycolipid transfer protein (GLTP) superfamily (6, 7, 8, 19, 20, 21).

In the GLTP superfamily, evolutionary modifications have led to the two-layer, all-α-helical GLTP-fold becoming adapted for binding SLs containing either phosphate or sugar headgroups, thus distinguishing two GLTP families. Examples of the phosphate headgroup-specific family include human ceramide-1-phosphate transfer protein (CPTP) and the plant CPTP ortholog, accelerated cell death-11 protein (ACD11) (22, 23), whereas glycolipid-specific members include human GLTP and phosphatidylinositol-4-phosphate adapter protein-2 (FAPP2) (19, 24, 25, 26, 27, 28, 29, 30). In all cases, the two-layer α-helical GLTP-fold binds the SL in “sandwich-like” fashion such that the initial phosphate or sugar residue of the SL headgroup is bound to the protein surface and the hydrocarbon chains are enveloped within a hydrophobic pocket.

Emerging information indicates that GLTP superfamily members can function *in vivo* as molecular sensors and/or presentation devices involved in lipid metabolic regulation and signaling processes. GLTP, FAPP2, CPTP, and ACD11 have been implicated in the *in vivo* regulation of SL homeostatic levels and intracellular distributions (22, 23, 25, 31, 32, 33, 34). In the case of human CPTP, siRNA-induced downregulation not only stimulates proinflammatory eicosanoid production but also triggers autophagy-dependent, inflammasome-mediated release of interleukin-1β and -18 and pyroptosis by macrophage-like surveillance cells (22, 33). CPTP intracellular docking sites include the *trans*-Golgi, a C1P production site by ceramide kinase, as well as the plasma and nuclear membranes (22). CPTP depletion leads to approximately fourfold *in vivo* elevations of intracellular C1P (mostly 16:0-C1P species) that accumulate in *trans*-Golgi-enriched membrane fractions and decrease in plasma-membrane-enriched fractions. The elevated C1P levels in the TGN stimulate arachidonic acid release and drive downstream production of proinflammatory eicosanoids (22), presumably reflecting activation of cytoplasmic phospholipase A_2_α *via* C1P binding to its C2 domain (35, 36).

GLTP superfamily members such as CPTP, ACD11, and GLTP lack known lipid-binding domains (LBDs) (*e.g.*, PH, PZ, C1, C2) that target various proteins to select phosphoglycerides in intracellular membranes (37, 38, 39, 40, 41, 42, 43). To determine whether CPTP and related GLTP homologs contain targeting motifs for specific phosphoglycerides embedded in membranes, we investigated the regulatory effects exerted by various phosphoinositides (PIPs) on SL transfer by CPTP, ACD11, or GLTP and their membrane partitioning. We focused on PIPs present in the *trans*-Golgi (*e.g.*, phospatidylinositol-4-phosphate; PI-4P) and plasma membrane (phospatidylinositol-4,5-bisphosphate; PI-(4,5)P_2_) due to earlier findings of CPTP enrichment at these intracellular sites (22). The data are consistent with PIP-specific headgroup interaction sites existing on CPTP but not GLTP that serve a dual role of enhancing SL transfer activity while also acting as preferred targeting/docking sites in specific membranes *in vivo*. Mapping of the PIP-selective motifs in C1P-specific GLTP-folds within membrane interaction regions reveals a role for the recently discovered ID-loop (α3-α4 helices connecting loop) (27).

## Results

### C1P transfer by CPTP is accelerated by PIP2 and PI-4P but not by PI-3P or PI

**Figure 1:**
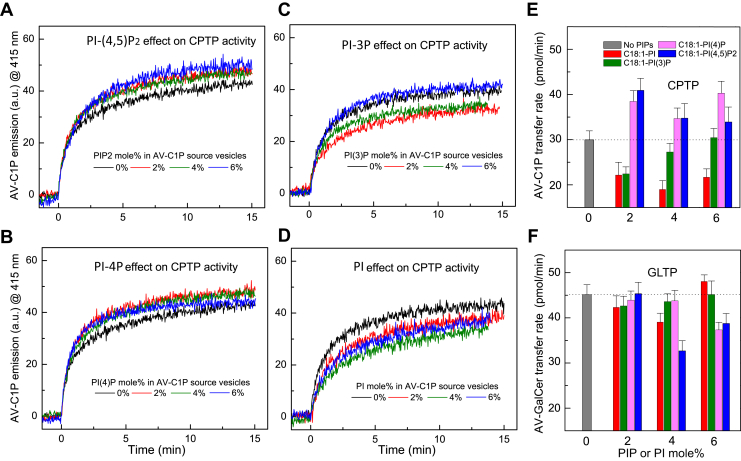
**PIP effects on SL transfer by human CPTP and human GLTP.** Traces in each panel show AV-SL emission intensity measured at 415 nm as a function of time resulting from FRET loss by AV-SL/Per-PC as AV-SL is transferred to POPC acceptor vesicles by CPTP (2 μg). *A*, PI-(4,5)P_2_ effects, *B*, PI-4P effects, *C*, PI-3P effects, and *D*, PI effects. *E* and *F*, summary of transfer rate changes induced by PI (*red*), PI-3P (*green*), PI-4P (*magenta*), PI-(4,5)P_2_ (*blue*), or no PIP (*gray*) for CPTP (*E*) and GLTP (*F*). SL transfer rates are expressed as pmol/min transferred from SL source to POPC vesicles as a function of different PIP amounts (mol%) in the SL source vesicles. Error bars, S.D.

To assess whether certain PIPs can activate the SL transfer activities of various GLTP superfamily members (human CPTP, plant CPTP-ACD11, human GLTP), we used an established fluorescence resonance energy transfer (FRET) approach that monitors the real-time kinetics of the complete SL transfer reaction, *i.e.*, SL uptake by protein from “SL-source” membrane vesicles and SL delivery by protein to “destination” membrane vesicles (44). A more complete description of the FRET assay is provided as Supporting information and illustrated in Fig. S1. Inclusion of various long-chain PIPs (PI-(4,5)P_2_, PI-4P, PI-3P) or PI in SL-source POPC vesicles was found to exert different effects on the SL intermembrane transfer rates catalyzed by CPTP and GLTP (Fig. 1) and by ACD11 (Fig. S2) at physiologic ionic strength. The PIP concentrations in the model membranes were kept low to mimic the physiological situation (45) and the buffer contained EDTA to block potential effects by polyvalent cations such as calcium (46). Notably, significant stimulation of C1P transfer rates occurred when 2, 4, or 6 mol% of PI-(4,5)P_2_ or PI-4P was present in the C1P source (donor) vesicles (Fig. 1, *A*–*E*). In contrast, PI-3P and PI failed to stimulate and inhibited, respectively, the C1P transfer activity of CPTP and exerted minimal effects on ACD11 (Fig. S2). Notably, GalCer transfer rates by GLTP were unaffected by PI and PI-3P as well as by 2 and 4 mol% PI-4P but were moderately decreased by 4 and 6 mol% PI-(4,5)P_2_ (Fig. 1*F*).

**Figure 2:**
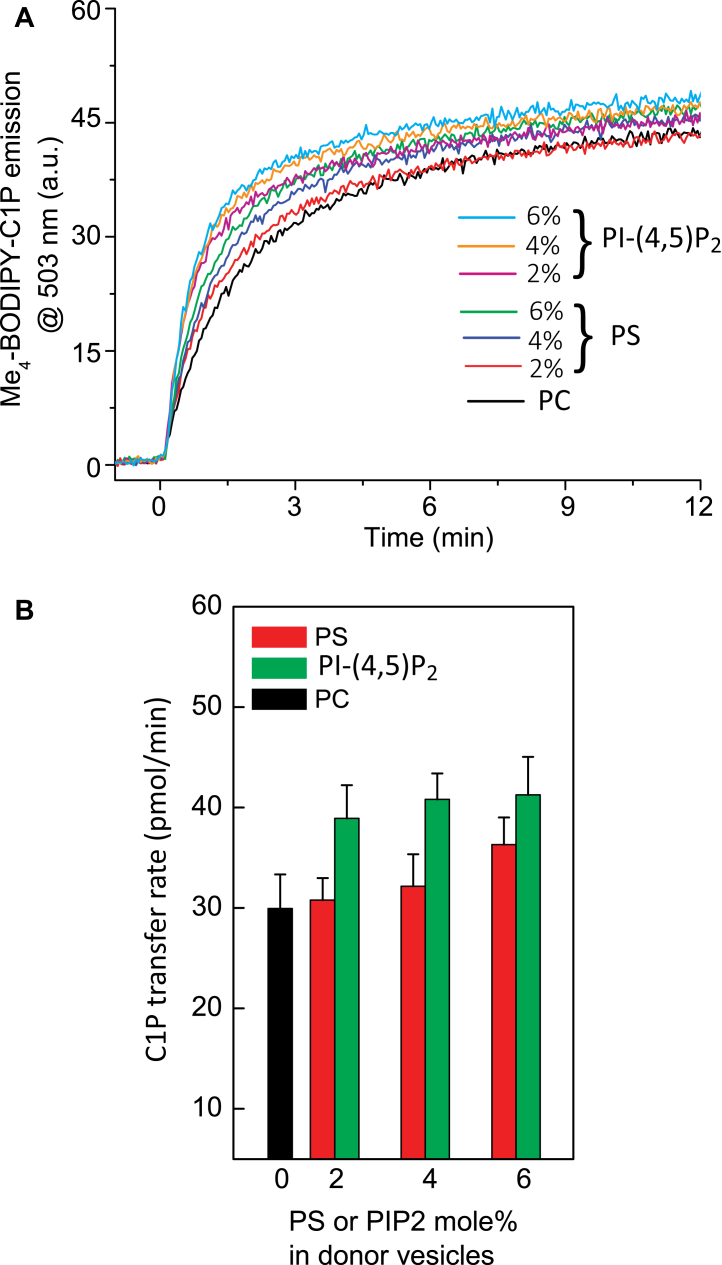
**CPTP transfer activity stimulation by PI-(4,5)P2 *versus* PS.***A*, traces show Me_4_-BODIPY-SL emission intensity measured at 503 nm as a function of time resulting from FRET loss by Me_4_-BODIPY-SL/C18-diI as Me_4_-BODIPY-SL is transferred to POPC acceptor vesicles by CPTP (2 μg). CPTP transfer activity stimulation by PI-(4,5)P2 is compared with that by PS. *B*, summary of CPTP transfer rates induced by PI-(4,5)P_2_ (*green*) *versus* PS (*red*). C1P transfer rates are expressed as pmol/min transferred from C1P source to POPC vesicles as a function of PI-(4,5)P_2_ or PS mol% in the SL source vesicles. Error bars, S.D.

To directly assess the ability of CPTP and ACD11 to interact with PI-(4,5)P_2_ and PI-4P, protein–lipid overlay assays were performed (47). Fig. S3 shows that both CPTP and ACD11 exhibit relatively strong binding interactions with PI-(4,5)P_2_ and PI-4P compared with various other anionic and zwitterionic phosphoglycerides, neutral lipids, and sulfatide. The observation of CPTP and ACD11 binding to phosphatidylserine (PS) and phosphatidic acid (PA) in the protein–lipid overlay assay, albeit moderate in intensity, is consistent with earlier findings for these two phosphoglycerides (48). In this previous study, SL transfer activity by CPTP and ACD11, but not GLTP, was found to be stimulated by membrane-embedded PS, but not by PA although no specific interaction site was identified. We therefore hypothesized that the CPTP membrane interaction region contains a specific binding site for targeting the PI-(4,5)P_2_ and/or PI-4P headgroups. These PIP headgroups presumably would act as a tethering/activation site to help favorably orient CPTP for C1P uptake during membrane interaction while the PIP acyl chains remain embedded in the membrane interior. To test this idea, we assessed whether C1P transfer by CPTP is stimulated by “soluble” PI-(4,5)P_2_ with short acyl chains (di-octanoyl- PI-(4,5)P_2_). We expected little or no activation by “soluble” PI-(4,5)P_2_ due to its much weaker anchoring in the POPC bilayer vesicle and its high aqueous solubility (cmc > 4 mM; (49)) compared with di-oleoyl PI-(4,5)P_2_. Indeed, replacing di-18:1 PI-(4,5)P_2_ with di-8:0-PI-(4,5)P_2_ in the SL-source vesicles resulted in no significant stimulation in C1P transfer rates by CPTP (Fig. S4). To test whether long-chain PI-(4,5)P_2_ is a better stimulator of CPTP than long-chain PS, we compared in side-by-side fashion. The data in Figure 2 show that PI-(4,5)P_2_ is a better stimulator of CPTP activity than PS at physiologically relevant PI-(4,5)P_2_ membrane concentrations (≤6 mol% in POPC).

**Figure 3:**
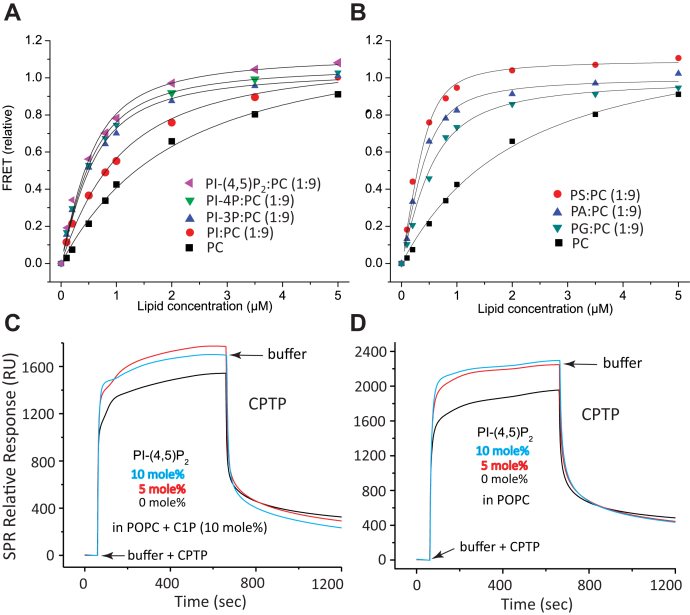
**Anionic phosphoglyceride effects on the membrane equilibrium binding by CPTP and PI-(4,5)P**_**2**_**effect on wtCPTP association/dissociation to/from POPC/C1P or POPC vesicles.** In *A* and *B*, traces show dansyl-PE emission intensity measured at 513 nm resulting from FRET by CPTP Trp/Tyr (2 μg; excit. 285) as a function of increasing concentration of POPC vesicles containing various PIPs and other anionic phosphoglycerides. In *C* and *D*, traces show surface plasmon resonance (SPR) data for CPTP adsorption and desorption to/from immobilized membrane vesicles of differing lipid composition. *C*, immobilized POPC vesicles containing C1P (10 mol%); *D*, immobilized POPC vesicles containing no C1P. In both panels, PI-(4,5)P_2_ amounts of 0 (*black trace*), 5 mol% (*red trace*), and 10 mol% (*cyan trace*) are shown for vesicles adsorbed to a Sensor Chip L1 (see Experimental procedures). wtCPTP injections are indicated by *arrows* and switches to buffer wash, by the *second arrows* in each trace.

To quantifiably assess and compare the extent to which the long-chain PIPs, PS, and other anionic phosphoglycerides impact the membrane-binding affinity of CPTP, we relied on FRET involving CPTP Tyr/Trp (energy donor) and POPC membrane vesicles containing dansyl-phosphatidylethanolamine (PE) (energy acceptor). Figure 3, *A* and *B* show the FRET responses produced by CPTP adsorption to POPC vesicles as a function of their concentration when containing equal amounts of PI-(4,5)P_2_, PI-4P, PI-3P, or PI (Fig. 3*A*) as well as PS, PA, or PG (Fig. 3*B*). Analyses of the binding isotherms resulted in the relative equilibrium binding affinity constants (*K*_d_) shown in Table 1. The *K*_d_ values for POPC vesicles containing PI-(4,5)P_2_, PI-4P, and PI-3P are six- to seven-fold lower than that for pure POPC vesicles (Table 1), whereas the *K*_d_ for POPC vesicles containing PI is only 2.3-fold lower. Interestingly, including PS and PA yielded relative *K*_d_ values even lower than the PIP values, which are comparable to that elicited by the presence of PG. The binding response for POPC vesicles containing PS and PA (relative to pure PC vesicles) agrees with earlier qualitative data showing that both PS and PA enhance membrane binding by plant CPTP, *i.e.*, ACD11, but only PS stimulates transfer activity of ACD11 and CPTP, but not GLTP (48).

**Table 1:** Relative equilibrium binding affinity of CPTP for POPC vesicles containing different anionic phosphoglycerides

Lipid	*K*_d_ (μM)	Relative to POPC
PI-(4,5)P_2_	0.26 ± 0.04	6.8
PI-4P	0.26 ± 0.03	6.8
PI-3P	0.29 ± 0.04	6.1
PI	0.75 ± 0.09	2.3
PS	0.07 ± 0.02	25.1
PA	0.11 ± 0.02	16.0
PG	0.24 ± 0.04	7.3
PC	1.76 ± 0.20	1.0

Anionic phosphoglycerides = 10 mol%.

To determine the impact of long-chain PI-(4,5)P_2_ on CPTP association and dissociation to/from PC membranes, surface plasmon resonance (SPR) analyses were performed. SPR provides real-time insights into protein adsorption and desorption to/from the membrane associated with SL uptake or release, *i.e.*, transfer “half-reactions.” The insights help understand the protein-mediated lipid transfer process, which involves: i) LTP association with the membrane; ii) lipid uptake by membrane-associated LTP; iii) LTP/cargo-lipid desorption from the membrane; iv) LTP/cargo-lipid association with acceptor membrane; v) LTP release of lipid cargo into the membrane; vi) lipid-free LTP desorption from acceptor membrane (19, 50, 51). Further complicating the situation is the proposed involvement of transient protein dimerization during membrane interaction (7). SPR experiments were initiated by introduction of CPTP into the flow cell after adsorbing and equilibrating the lipophilic sensor chip with vesicles of differing lipid composition. As shown in Figure 3, *C* and *D*, inclusion of 5 or 10 mol% PI-(4,5)P_2_ in POPC vesicles significantly enhanced CPTP adsorption both in the presence or in the absence of C1P.

### Location of the PI-(4,5)P_2_ interaction site(s) on CPTP

**Figure 4:**
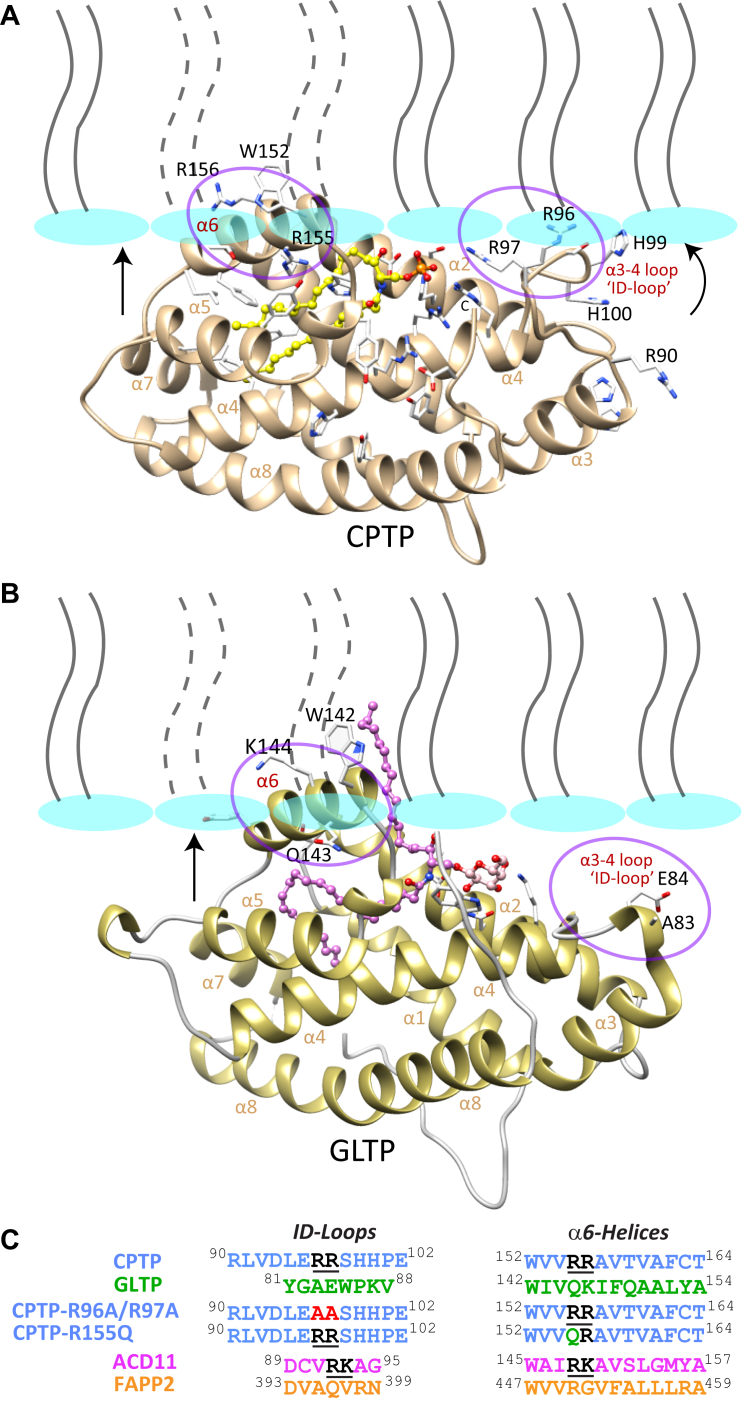
**OPM-based structural model for membrane interaction by CPTP.***A*, orientation of peripheral protein during membrane interaction (OPM) modeling for CPTP complexed with 16:0 C1P (PDB: 4k84; 1.90 Å resolution) reveals favorable positioning of the α3-α4 helices connecting loop (“ID-loop”) and the α6-helix and their respective R96/R97 and R155/R156 di-Arg motifs for acting as potential interaction sites for PIP headgroup binding. *B*, OPM modeling for GLTP complexed with 24:1 GalCer (PDB: 4euk; 1.85 Å resolution), which is not stimulated by PIPs, has A83/E84 in the α3-α4 helices connecting loop (“ID-loop”) and Q143/K144 in α6-helix at structurally equivalent positions as diArg in CPTP. *C*, sequence alignments for the di-Arg motif regions of the ID-loop and α6-helix in CPTP compared with the same regions in GLTP, ACD11, and FAPP2. Alignments are derived from comparisons of their crystal structures (28).

Di-Arg motifs are known to function as PI-(4,5)P_2_ interaction sites in the Rho GTPase, Cdc42 (52) and as PI-3P and PI-(3,5)P_2_ interactions sites in the PROPPIN Atg18 (53). Human CPTP contains two di-Arg motifs but GLTP, which is not activated by PIP2, has none. The two di-Arg motifs in wtCPTP are located near the C1P-binding site that resides within the membrane interaction region of the protein. To assess the potential for these two di-Arg motifs to function as PI-(4,5)P_2_ interaction site(s) on human CPTP, we examined CPTP docking to membranes as modeled by the Orientation of Proteins in Membranes (OPM) approach (54). Figure 4*A* illustrates membrane interaction by CPTP prior to sphingolipid uptake with respect to overall protein orientation and penetration as well as initial docking regions. The embedding of α-helix 6 in the membrane is well supported by experimental data (7, 19, 29, 55, 56). One di-Arg motif (R155-R156) of CPTP is located in α-helix 6 near Trp152, which has previously been identified as a key residue for membrane interaction by GLTP superfamily members (22). The OPM-guided model of CPTP orientation during interaction with the bilayer interface reveals that the R155-R156 diArg motif in α-helix 6 is favorably positioned for interaction with the PI-(4,5)P_2_ headgroup in the bilayer surface (Fig. 4*A*).

The other di-Arg motif (R96-R97) is located within the α-3/α-4 helices connecting loop, also known as the ID-loop (27). The OPM-guided model indicates that the R96-R97di-Arg motif in the α3-α4 helix connecting loop of CPTP is also reasonably well-positioned for interaction with the PI-(4,5)P_2_ headgroup. The ID-loops of GLTP superfamily members vary in length (Fig. 4*B*) and conformation but generally have stabilizing intraloop interactions (27). In this regard, the α3-α4 helix connecting loop (ID-loop) of CPTP and ACD11 (Fig. S5) appears better suited for PI-(4,5)P_2_-mediated membrane interaction than that of GLTP (Fig. 4*C*). Notably, di-Arg motifs do not occur in the ID-loop or α-helix 6 of GLTP but a single Lys residue is present in the α-6 helix.

To experimentally test for involvement of the two CPTP di-Arg motifs in binding to PI-(4,5)P_2_, we generated ID-loop mutant R96A/R97A as well as helix-6 mutant R155A-/R156A (Fig. 4*C*). Yet, we were able to successfully express and purify only CPTP R96A/R97A due to insolubility issues for CPTP R155A/R156A. To circumvent this issue, Arg155 was mutated to glutamine (Q), whereas Arg156 remained unaltered to mimic the situation in GLTP, which shows no transfer activity stimulation by PI-(4,5)P_2_ and contains α-helix 6 residues, Q145-K146 at the structural positions corresponding to R155-R156 in CPTP (Fig. 4*B*). Residues A83-E84 of the GLTP α3/α4 helix connecting loop (ID-loop) are located at structural positions corresponding to R96/R97 of CPTP (27).

**Figure 5:**
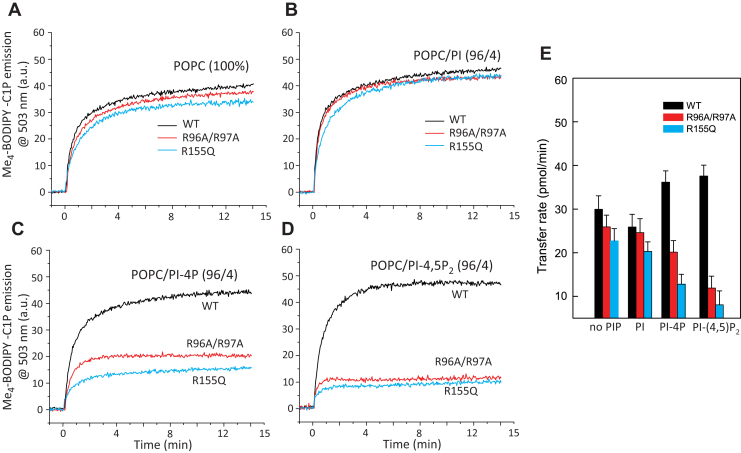
**Mutation mapping identifying di-Arg motifs as PIP sites affecting CPTP-mediated C1P Transfer.** Traces in each panel show BODIPY-SL emission intensity at 503 nm as a function of time (due to BODIPY-SL/C18-DiI FRET loss) when BODIPY-SL transfers to POPC vesicles. SL donor vesicles compositions are: *A*, POPC; *B*, POPC/PI (96:4); *C*, POPC/PI-4P (96:4); and *D*, POPC/PI-(4,5)P_2_ (96:4). In each panel, wtCPTP (*black trace*), CPTP-R96A/R97A (*red trace*), and CPTP-R155Q (*blue trace*) are compared. Data for 2 mol% and 6 mol% POPC/PI-(4,5)P_2_ are provided in Fig. S6. *E*, Comparison of C1P transfer rates for data shown in panels *A*, *B*, *C*, and *D*. The SL transfer rates are expressed as pmol/min transferred from SL source POPC vesicles. Error bars, S.D.

Assessment of the CPTP-R96A/R97A and CPTP-R155Q transfer activities of BODIPY-C1P to POPC acceptor vesicles from donor vesicles containing no PIP or 4 mol%, PI, PI-4P, or PI-(4,5)P_2_ is shown in Figure 5. The mutations produced measurable, moderate effects on the baseline transfer activities from POPC donor vesicles lacking PIPs (Fig. 5*A*). A similar impact of the mutations on C1P transfer was observed when POPC donor vesicles contained 4 mol% PI (Fig. 5*B*). However, mutation of the di-Arg site in either α-helix 6 or in the α3-α4 helix connecting loop (ID-loop) not only eliminated the activation effects observed for 4 mol% PI-4P and PI-(4,5)P_2_ on wtCPTP but also dramatically slowed C1P transfer from POPC vesicles containing these PIPs (Fig. 5, *C* and *D*) with a stronger effect exerted by PI-(4,5)P_2_. Measurements performed using donor vesicles containing either 2 or 6 mol%, PI, PI-4P, or PI-(4,5)P_2_ (Fig. S6) resulted in similar trends albeit moderately weaker or stronger in absolute magnitude compared with 4 mol % PI-(4,5)P_2_.

**Figure 6:**
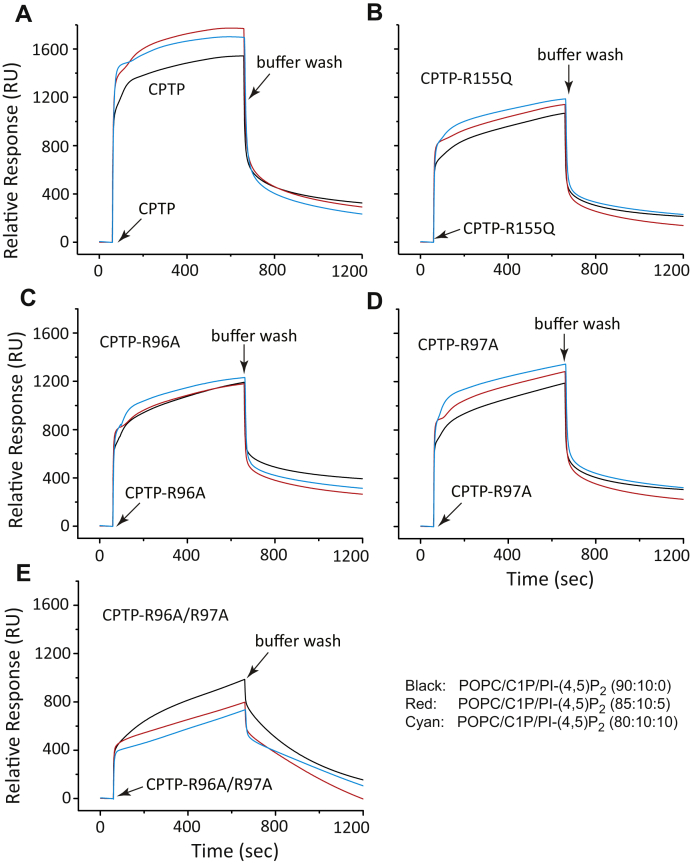
**Mutation mapping showing di-Arg motif involvement in CPTP partitioning to POPC/C1P vesicles containing PI-(4,5)P**_**2**_**.** SPR assessments of CPTP mutant adsorption and desorption to/from immobilized membrane vesicles of differing lipid composition. *A*, wtCPTP; *B*, CPTP-R155Q; *C*, CPTP-R96A; *D*, CPTP-R97A; *E*, CPTP-R96A/R97A. Injections are indicated by the *first arrows* and switches to buffer wash, by the *second arrows* in each trace. In all panels, PI-(4,5)P_2_ amounts of 0 (*black trace*), 5 mol% (*red trace*), and 10 mol% (*cyan trace*) are shown for vesicles adsorbed to the Sensor Chip L1 (see Experimental procedures for details).

To directly assess the extent to which the mutations impacted protein association and dissociation with PC membranes, SPR analyses were performed. We focused on PI-(4,5)P_2_ because of the stronger stimulatory response elicited by this PIP and because of our previous findings showing CPTP transfer of C1P from the *trans*-Golgi to the plasma membrane (22) where PI-(4,5)P_2_ is known to localize intracellularly (45). Figure 6 shows the SPR responses for wtCPTP and various ID-loop or helix 6 mutants. Enhanced association by wtCPTP was observed when the immobilized POPC/C1P vesicles contained PI-(4,5)P_2_ (Fig. 6*A*). Notably, when the immobilized POPC/C1P vesicles lacked PI-(4,5)P_2_, replacement of Arg in either the ID-loop (R96A, R97A, and R96A/R97A) or helix-6 (R155Q) resulted in diminished CPTP association compared with that of wtCPTP (Fig. 6, *A*–*E*; black data curves). This finding is not surprising and likely reflects the well-established role for Arg residues in nonspecifically enhancing protein–membrane interactions *via* “snorkeling” involving side-chain guanidinium interactions with the negatively charged phosphate residues of phosphoglycerides such as PC (57, 58, 59). Nonetheless, the enhanced association response for POPC bilayers containing PI-(4,5)P_2_ remained strong for CPTP-R155Q and CPTP-R97A (Fig. 6, *B* and *D*) but was significantly diminished for CPTP-R96A (Fig. 6*C*). In contrast, association of the CPTP double mutant (R96A/R97A) with immobilized POPC/C1P vesicles containing PI-(4,5)P_2_ was clearly negatively impacted (Fig. 6*E*). Similar overall trends also were observed for both wtCPTP and the mutants when immobilized POPC vesicles lacked C1P but contained PI-(4,5)P_2_ (Fig. S7). Taken together, the data support involvement of both di-Arg sites within the CPTP membrane interaction region for mediating binding with PI-(4,5)P_2_ embedded in POPC membranes. The CPTP di-Arg site functionality occurs regardless of the presence or absence of C1P. Maximum stimulation of CPTP transfer activity by PI-(4,5)P_2_ requires both Arg residues of the R96-R97 site in the ID-loop.

## Discussion

Our investigation provides evidence for PIP regulatory sites existing in the membrane-interaction region of CPTP, a single-domain GLTP superfamily member that transfers C1P between membranes (6, 7, 8, 22, 23). Previously, identification of lipid regulators controlling the membrane interaction of 4-phosphate adaptor protein-2 (FAPP2), a multidomain GLTP superfamily member that transfers GlcCer from the *cis*-Golgi, focused on its N-terminal pleckstrin homology (PH) domain that interacts with PI-4P without attention to its GLTP homology (GLTPH) domain (25, 60). Thus, the possibility for PIP regulation *via* direct interaction with GLTPH domain or with single-domain GLTP superfamily members has remained unclear.

Our data show that PI-4P and PI-(4,5)P_2_, but neither PI-3P nor PI, stimulate the SL transfer activity of human CPTP and plant ACD11 C1P-specific GLTP-folds at physiologic ionic strength and in the absence of Ca^2+^. The stimulation by PI-4P and PI-(4,5)P_2_ is not duplicated for human GLTP, a glycolipid-specific GLTP-fold. X-ray structures of CPTP and ACD11 complexed with C1P show a conserved cationic Arg/Lys triad interacting with the C1P headgroup and a Asp-His “clasp” interacting with the ceramide amide moiety along with additional Arg and Lys residues in the membrane interaction region surrounding the SL headgroup recognition site (6, 7, 22, 23). In addition to protein structural features and simple charge–charge effects, membrane-related factors need also to be considered when evaluating lipid transfer processes. CPTP interacts only with PIPs and C1P located in the outer leaflet of the bilayer vesicle. The membrane interactions by GLTP homologs occur in a moderately penetrating and minimally perturbing manner that leaves the sphingolipid pool in the inner leaflet of the vesicle bilayer inaccessible to protein (50, 51, 61, 62). Thus, neither GLTP nor CPTP nor ACD11 can access the inner bilayer leaflet or promote transbilayer migration of their target SLs. Spontaneous transbilayer migration of C1P is highly restricted due to the unfavorable energetics of moving the phosphate polar headgroup through the nonpolar hydrocarbon matrix.

In mammals, C1P is produced anabolicly by ceramide kinase at the *trans*-Golgi cytosolic face (63, 64) and then is transported to other intracellular sites such as the plasma membrane cytosolic surface (22). Accordingly, C1P is localized initially only in the C1P source (donor) vesicles and not in the destination (acceptor) vesicles. *In vivo*, certain PIPs reside in specific intracellular membranes that face the cytosol (45, 65, 66, 67, 68), where they can potentially be targeted by various peripheral amphitropic proteins including CPTP or GLTP. PI-4P resides in the *trans*-Golgi, plasma membrane, and endosome/lysosome (66). PI-(4,5)P_2_ localizes predominantly to the plasma membrane (45). PIP concentrations in POPC-SL source vesicles were kept low (≤10 mol %) to mimic the PIP physiological situation.

The selective *stimulation* of C1P transfer activity by anionic PI-4P and PI-(4,5)P_2_, but not by PI-3P or PI, prompted us to consider the existence of a PIP-headgroup specific surface-binding site(s) located within the CPTP membrane interaction region. Because replacement of di-18:1 PI-(4,5)P_2_ with short-chain (di-8:0) “soluble” derivatives fails to stimulate C1P transfer by CPTP, the PIP headgroup needs to remain firmly associated with the C1P-source membrane to activate C1P transfer by CPTP. It is noteworthy that the equilibrium binding constants for CPTP with POPC vesicles containing various anionic phosphoglycerides do not correlate with their capacity to stimulate or slow SL transfer activity. For instance, the equilibrium membrane-binding constant for POPC vesicles containing PI-(4,5)P_2_ is three- to four-fold larger than that for POPC vesicles containing PS (Table 1). Yet, PI-(4,5)P_2_ is the better stimulator at low membrane concentrations. This situation likely reflects the complex, multistep mechanism of CPTP action. It also is worth remembering that the FRET efficiency is affected by both distance and orientation of the fluorophores. In CPTP, two of three Trp residues are located close to the C1P-binding site. We conclude that the enhanced partitioning driven by PI-3P, PI, PA, and PG leaves CPTP in unfavorable membrane orientations that slow C1P uptake/transfer. By contrast, more favorable orientations that help enhance C1P uptake/transfer and (increases FRET efficiency) are elicited by PS, PI-(4.5)P_2_, and PI-4P. We propose that these phosphoglycerides function as membrane tethering sites that engage and orient CPTP in ways that optimize function while also possibly helping to target certain intracellular locations. This thinking led us to look beyond simple CPTP surface charge near the C1P-binding site and consider directly testing for the existence of a PI-(4,5)P_2_-headgroup specific surface-binding site within the membrane interaction region of CPTP.

**Figure 7:**
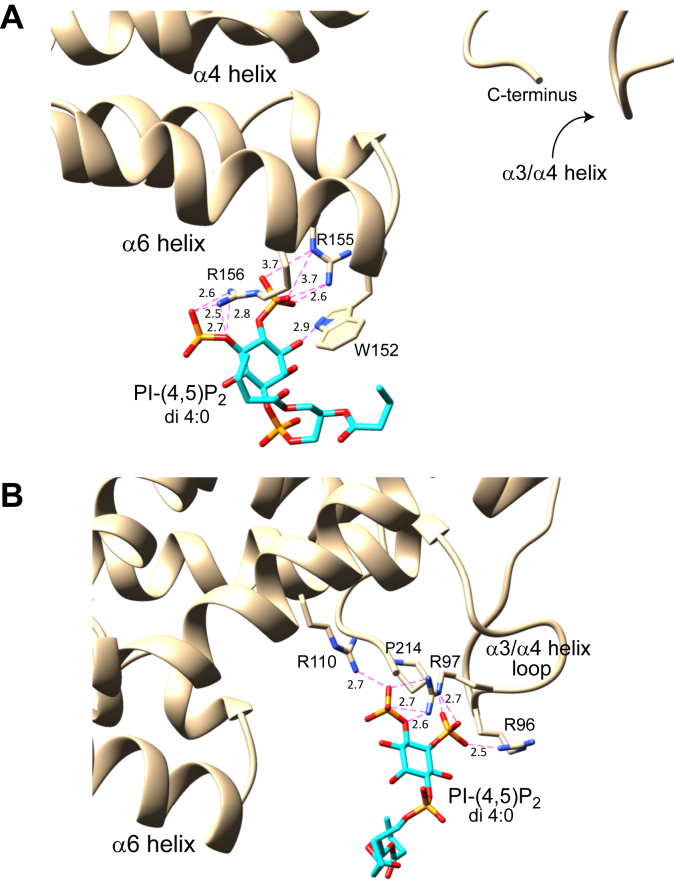
**Haddock modeling of PIP2 interaction with CPTP di-Arg sites.***A*, Haddock model of PI-(4,5)P_2_ interaction with the R155/R156 motif of CPTP. *B*, Haddock model of the PI-(4,5)P_2_ interaction with the R96/R97 motif of CPTP. The representative complex structures come from the top cluster (with the lowest HADDOCK score), which was Cluster 1 for R555/R156 and Cluster 3 for R96/R97 (see Fig. S9).

Mapping of the PI-(4,5)P_2_ headgroup-binding sites by mutational functional analyses, OPM membrane modeling, and HADDOCK modeling indicate involvement of two di-Arg sites within the CPTP membrane interaction region. Whereas OPM helps identify the molecular regions in peripheral proteins (*e.g.*, helix 6 and the ID-loop of CPTP) that interact with membranes (Fig. 4 & Fig. S5), HADDOCK modeling provides a molecular picture of how the di-Arg motifs in helix 6 and the ID-loop are likely to engage the phosphorylated inositol ring of PI-(4,5)P_2_ (Fig. 7). It is noteworthy that these di-Arg motifs are lacking in α-helix 6 and in the ID-loop (α3-α4 helix connecting loop) of human GLTP, which is not activated by PI-(4,5)P_2_. Haddock modeling is an information-driven, flexible docking approach that differs from *ab initio* docking methods by encoding information from identified or predicted protein interfaces in ambiguous interaction restraints (AIRs) to drive the docking process (69, 70). HADDOCK is able to address various modeling problems including protein–ligand, protein–protein, and protein–nucleic acid complexes. In our case, Haddock modeling reveals specific interactions by the PI-(4,5)P_2_ headgroup and acyl chains oriented favorably for membrane embedding (Fig. 7).

### Novel features of CPTP PIP2 motifs compared with lipid-binding domains (LBDs)

In some respects, the CPTP PIP interaction sites function analogously to LBDs. Such domains (*e.g.*, C1, C2, PH, PX, FYVE) exist as modular structural elements within multidomain proteins. LBDs target the phosphoglyceride headgroup and contain no hydrophobic pocket for enveloping the lipid aliphatic chains. This arrangement keeps the lipid chains embedded in the membrane while the protein interacts with the phosphoglyceride headgroup. LBDs function as autonomous membrane docking modules that help selectively tether various peripheral, amphitropic proteins to select intracellular membrane sites while sometimes regulating nearby catalytic domains (37, 38, 39, 40, 41, 42, 43). In contrast, single-domain CPTP harbors both PI-(4,5)P_2_ docking sites very near to its C1P cargo binding site, enabling coincident site functionality within the CPTP membrane interaction region. The somewhat differing responses of each PI-(4,5)P_2_ headgroup-binding site to mutation further suggest that cooperativity may play a role in optimally orienting CPTP for C1P uptake/release. The close proximity of the PIP targeting motifs to the C1P-binding site raises the possibility that CPTP interaction with long-chain PIPs embedded in C1P-containing membranes could facilitate protein conformational changes that enhance C1P uptake or release. The preceding ideas will need future comprehensive study.

What is clear is that PI-(4,5)P_2_ interacts with CPTP in a fundamentally different way compared with LBDs and other peripheral proteins. While cationic residues (Arg, Lys) play significant roles in all such proteins, there is no conserved structural arrangement of these residues, which generally are located at junctures of protein structural elements. Fig. S8 illustrates by showing the structures of the PI-(4,5)P_2_-binding sites involving β-strand crevices such as that in rabphilin C2A domain (β3/4 strand residues) and the two sites in Arf GAP ASAP1 PH domain (β1/2 & β3/4 strand and β6/7 loop residues = canonical site; β1/2 opposite side residues adjacent to β5/6 loop residues = atypical site). Helix clusters involving three and two helices that favorably converge residues for PI-(4,5)P_2_ binding occur in ENTH epsin and metavinculin and are shown in Fig. S8, *C* and *D*, respectively. These situations contrast that of the CPTP PI-(4,5)P_2_ interaction sites where its two di-Arg motifs are self-contained within single elements (α6-helix or α3/α4 helix connecting loop) that each reside within the protein’s membrane interaction region.

### Physiological perspectives

The discovery of PI-(4,5)P_2_- and PI-4P-induced enhancement of C1P-specific GLTP homolog action provides insights into how CPTP and ACD11 could be targeted to and site-specifically stimulated by certain membranes in animal and plant cells, respectively. Intracellularly, PIPs occur in the cytosol-facing surfaces of the plasma membrane, endosomes, and Golgi enabling docking and activation by important cytoplasmic signaling and fusogenic proteins with specific PIP-binding domains (45, 71). PI-4P recruits not only coat proteins and accessory factors required for vesicular transport from the Golgi but also other lipid-binding/transfer proteins such as OSBP, CERT, and FAPP2, which contain PH domains that bind to PI-4Ps, to mediate their Golgi localization. PI-(4,5)P_2_ aids in the activation of plasma membrane channels/transporters, serves as a precursor for the generation of second messengers, and functions as a plasma membrane recruiter for cytosolic peripheral proteins such as those shown in Fig. S7 (67, 72, 73, 74, 75, 76). Human CPTP, a regulator of proinflammatory eicosanoid production, autophagy, inflammasome assembly, and pyroptosis, and ACD11, a regulator of accelerated cell death in plants, can now be added to this growing list of amphitropic peripheral membrane proteins. Our findings support earlier observations (22, 33) showing CPTP enrichment at the *trans*-Golgi and on the cytoplasmic surfaces of endosomes and plasma membrane, sites where PIPs also reside.

## Conclusions

Significant stimulation of human and plant C1P-specific lipid transfer proteins, at physiological ionic strength and in the absence of divalent cations (*e.g.*, calcium), occurs when certain PIPs (PI-4P and PI-(4,5)P_2_), but not PI-3P nor PI, are embedded in POPC bilayer vesicles. Notably, “soluble” PIPs that do not remain firmly embedded in the bilayer matrix produce no stimulatory effect on C1P transfer. By contrast, glycolipid-specific human GLTP activity is not activated by any of the three PIPs tested (PI-4P, PI-(4,5)P_2_). CPTP binding to the PI-(4,5)P_2_ headgroup involves di-Arg motifs located in α-helix 6 and in the α3-α4 helices connecting loop (“ID-loop”) of the GLTP-fold. While α-helix 6 involvement in membrane docking by GLTP superfamily proteins is well established, elucidation of a unique membrane interacting role for the ID-loop of C1P-specific GLTP superfamily members is novel. The binding sites for PI-(4,5)P_2_ (and possibly PI-4P) function are proposed to help optimally orient and tether CPTP (and ACD11) on the membrane surface for uptake and release of C1P during the SL transfer process. The findings are important because C1P-specific lipid transfer proteins are known regulators of inflammation and programmed cell death (8), although potential mechanisms by which these proteins can be targeted to certain intracellular destinations have remained largely unknown until now.

## Experimental procedures

1-Palmitoyl-2-oleyl-*sn*-glycero-3-phosphocholine (POPC), 1,2-dioleoyl-*sn*-glycero-3-phospho-(1'-myo-inositol) (PI); 1,2-dioleoyl-*sn*-glycero-3-phospho-(1'-myo-inositol-4'-phosphate) (di18:1 PI-4P); 1,2-dioctanoyl-*sn*-glycero-3-phospho-(1'-myo-inositol-4'-phosphate) (di8:0 PI-4P); 1,2-dioleoyl-*sn*-glycero-3-phospho-(1'-myo-inositol-4',5'-bisphosphate) (di18:1 PIP2), and 1,2-dioctanoyl-*sn*-glycero-3-phospho-(1'-myo-inositol-4',5'-bisphosphate) (di8:0 PIP2) were purchased from Avanti Polar Lipids and used without further purification. Lipid labeled with 3-perylenoyl (Per), anthrylvinyl (AV), or 4,4-difluoro-1,3,5,7-tetramethyl-4-bora-3a,4a-diaza-*s*-indacene (Me_4_-BODIPY) fluorophores (*e.g.*, Per-PC, AV-C1P, AV-GalCer, Me_4_-BODIPY-C1P, Me_4_-BODIPY-GalCer) were synthesized by lyso-lipid reacylation with omega-labeled 9-(3-perylenoyl)-nonanoyl, (11*E*)-12-(9-anthryl)-11-dodecenoyl, or 15-(Me_4_-BODIPY)-pentadecanoyl chains followed by purification (77, 78, 79). 1,1'-di-octadecyl-3,3,3',3'-tetramethylindocarbocyanine perchlorate (DiIC_18_) was purchased from Molecular Probes of Thermo Fisher Scientific.

### Recombinant protein purification

Cloning, expression, and purification of ACD11, CPTP, and GLTP have been described previously (22, 23, 80, 81, 82). Briefly, the open reading frames (ORFs) for human *CPTP* (GenBank JN542538 & NP_077792.2), *Arabidopsis acd11* (NCBI NP_181016.1) and human *GLTP* (GenBank AF209704) were ligated into pET-28 vector (kanamycin-resistant; Invitrogen) modified with small ubiquitin-like modifier (SUMO) protein ORF, which were then used to transform BL21 (DE3)-pLysS cells for expression of proteins N-terminally tagged with 6xHis-SUMO (22, 23). Transformed cells were grown in Luria-Bertani medium at 37 °C for 6 h, induced with 0.1 mM IPTG, and then incubated 16 to 20 h at 15 °C. Affinity protein purification from soluble lysate was accomplished by Ni-NTA affinity chromatography. Cleavage of N-terminal 6xHis-SUMO tag was carried out with SUMO protease, Ulp1, overnight at 4 °C. Affinity repurification by Ni-NTA chromatography followed by FPLC gel filtration chromatography (HiLoad 16/60 Superdex-75 prep grade column; GE Healthcare), equilibrated with buffer containing 25 mM Tris-HCl, pH 8.0, 100 mM NaCl and 1 mM DTT, yielded proteins with native sequences. Pooled peak fractions were concentrated by centrifugal concentrators (Vivaspin; 10 kDa cutoff). Protein purity was confirmed by SDS-PAGE (81) before flash freezing the pure proteins in buffer containing 50% glycerol and storing at −20 °C.

### Protein-mediated sphingolipid intermembrane transfer

Real-time intermembrane transfer rates of fluorescent sphingolipids by CPTP, ACD11, and GLTP were obtained by Förster resonance energy transfer (FRET) using a SPEX FluoroLog3 spectrofluorimeter (Horiba Scientific), with excitation and emission band passes of 2 nm and a stirred (∼100 rpm), temperature-controlled (25 °C ± 0.1 deg. C) sample cuvette holder (44, 48, 61). All fluorescent lipids were localized initially to the sphingolipid-source (donor) POPC vesicles formed by rapid ethanol injection. Excitation of AV- (370 nm) or BODIPY-sphingolipid (460 nm) results in minimal emission at 415 nm or 503 nm, respectively due to resonance energy transfer to nearby Per-PC or C18-DiI, respectively. Addition of approximately tenfold excess of sonicated POPC acceptor vesicles or POPC/DHPC bicelles produces little change in fluorescence signal, yielding a “no protein” baseline response for spontaneously transferred AV-sphingolipid, which is very slow (83, 84). Protein addition triggers a sudden, hyperbolic increase in AV or BODIPY emission intensity (415 nm or 503 nm, respectively) reflecting the FRET decrease due to protein transport of fluorescent sphingolipid to receiver (acceptor) vesicles and separation from nontransferable Per-PC or C18-DiI lipids in sphingolipid-source vesicles. The use of the two different FRET fluorophore donor/acceptor pairs shows that the structural features of any one set of fluorophore probe pairs are not responsible for the basic experimental outcomes. Maximum sphingolipid transfer, Δ*F*, is the difference in emission intensity in the absence and presence of protein late in the kinetic time course (>15 min) and arises from the fluorescent sphingolipid present in the outer leaflets of the sphingolipid-source vesicles and accessible to the protein. Addition of Tween-20 detergent after extended incubation provides a measure of maximum intensity achievable at “infinite” fluorophore separation. Nonlinear regression analyses using ORIGIN 7.0 software enable quantification of the initial lipid transfer rate, ν_0_, for the first-order exponential transfer process. Standard deviations were calculated at 95% confidence interval. R2 values for all estimates were >0.96.

### Vesicle preparation

Acceptor POPC vesicles and donor vesicles composed of POPC (97.5 mol%), AV-lipid (1 mol%), and Per-PC (1.5 mol%) or POPC (97.5 mol%), BODIPY-15-GalCer (1 mol%), and DiI-C18 (1.5 mol%) were prepared as described in (61). Acceptor vesicle diameter averaged 25 to 30 nm. The final acceptor vesicle concentration in the FRET lipid transfer assay was ∼85 μM, which was tenfold higher than that of the donor vesicles. Buffer contained 10 mM K phosphate (pH 6.6), 150 mM NaCl, and 0.2% EDTA.

### FRET equlibrium binding affinity measurements

Partitioning of CPTP to membrane vesicles was monitored by FRET using Trp/Tyr emission of CPTP as the energy donor and POPC vesicles containing dansyl-PE (2 mol%) and (10 mol% of the phospholipid to be tested) as energy acceptors as described in (50). Vesicles were formed by mixing the POPC, dansyl-PE, and other lipids, drying under a stream of nitrogen and placing under vacuum for ∼2 h, before suspending in ethanol. Binding reactions included CPTP (0.5 μM) and various amounts of vesicles formed by rapid ethanol injection (concentration from 0.1 to 5 μM) into 2 ml of stirred buffer containing 10 mM K phosphate (pH 6.6), 150 mM NaCl, and 0.2% EDTA. FRET measurements were performed at 25 °C in a temperature-controlled (±0.1 °C) cuvette (NesLab RTE-111, Thermo Fisher) using a SPEX FluoroLog-3 spectrofluorimeter (Horiba Scientific). Excitation and emission wavelengths were 284 nm and 513 nm with band-pass settings of 5 and 10 nm, respectively. FRET was calculated as (I_obs_ − I_min_)/(I_max_ − I_min_), where I_min_ is the dansyl emission in the absence of vesicles and I_max_ is the maximal energy transfer obtained from the binding curve. FRET data were plotted as relative fluorescence signal *versus* PC concentration of the vesicles and fit to the equation described in (50).

### Surface plasmon resonance of protein partitioning to membranes

Assays were performed using a Biacore T200 system (GE Healthcare Bio-Sciences Corp). POPC/SL/phosphoinositide vesicles (1 mM) containing 10 mol% SL were prepared by brief sonication centrifuged (13,000 rpm × 10 min) and then captured on a Sensor Chip L1 to a final surface density of 3000 to 6000 response units to establish the baseline prior to protein addition. Injections of proteins or buffer were performed at 5 μl/min flow rates as recently described in (48). The setup and wash conditions used for monitoring protein adsorption/desorption were similar to those described in (27).

### OPM and HADDOCK modeling

The OPM computational approach was used to identify residues involved in the initial docking of CPTP, ACD11, and GLTP with the membrane interface as shown in Figure 4 and Fig. S5 (54). HADDOCK modeling was used to gain insights into the interaction between PI-(4,5)P_2_ and CPTP at the molecular level by docking di-4:0 PI-(4,5)P_2_ with the di-Arg motifs in α-helix 6 or the α-3/4 helices connecting loop using HADDOCK 2.4 available online (https://wenmr.science.uu.nl/haddock2.4; (69, 70)). HADDOCK-calculated docking interfaces are based on experimental knowledge in the form of AIRs (see Fig. S9). R96/R97 or R155/R156 was designated as active amino acid residues based on experimental data and passive amino acid residues were automatically generated by the program. Illustration, visualization, and analyses of the docked complexes were provided by UCSF Chimera (85) for their interaction studies.

## Data availability

All data are contained within the article and the supporting information.

## Supporting information

This article contains supporting information.

## Conflict of interest

The authors declare that they have no conflicts of interest with the contents of this article.
