# Supplementary material for: Ceramide-1-phosphate transfer protein (CPTP) regulation by phosphoinositides
Source: J Biol Chem. 2021 Mar 26;296:100600. doi: 10.1016/j.jbc.2021.100600 (PMC8091061; doi:10.1016/j.jbc.2021.100600)
Supplement: Supplemental Figures S1–S9 [file mmc1.pdf]

Ceramide-1-phosphate transfer protein (CPTP) regulation by phosphoinositides[#]

Yong-Guang Gao[1], Xiuhong Zhai[1,¶], Ivan A. Boldyrev[2], Julian G. Molotkovsky[2],
Dinshaw J. Patel[3], Lucy Malinina[1], Rhoderick E. Brown[1,*]

[1]Hormel Institute, University of Minnesota, 801 16th Ave NE, Austin, MN 55912

[2]Shemyakin-Ovchinnikov Institute of Bioorganic Chemistry, Russian Academy of Sciences, 117997 Moscow, Russian Federation

[3]Structural Biology Program, Memorial Sloan-Kettering Cancer Center, New York, NY 10065

Supporting Information

Figure S1

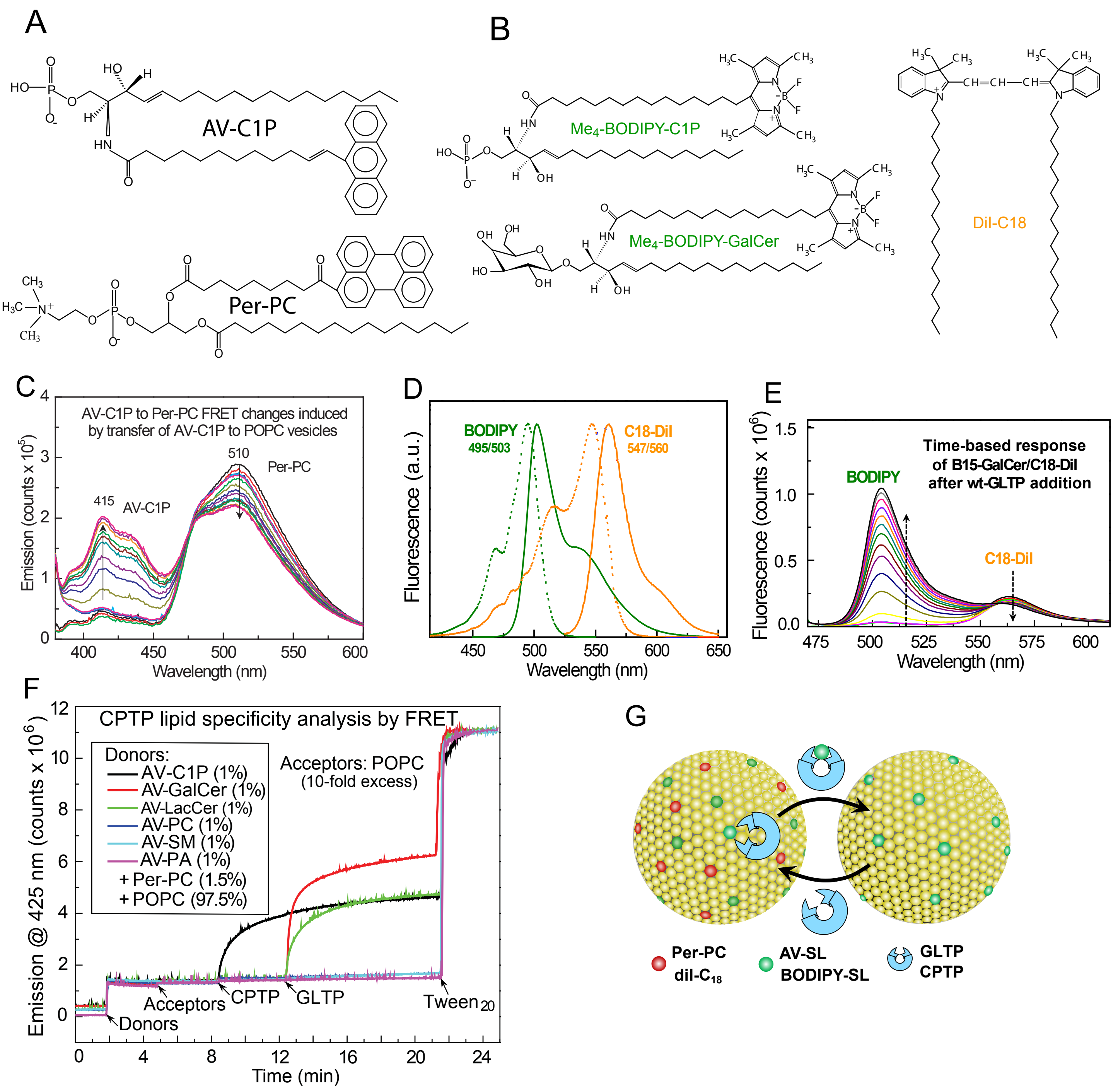

Figure S1. Lipid transfer measurement by lipid transfer proteins using FRET. A) Structures of AV-C1P a FRET energy donor that is transferred by CPTP, and nontransferable FRET energy acceptor (3-perylenoyl phosphatidylcholine). **B)** Structures of Me4-BODIPY-C1P and Me4-BODIPY-GalCer FRET energy donors that are transferred by CPTP and GLTP, respectively, and nontransferable FRET energy acceptor (C18-DiI). **C)** FRET signal changes induced by CPTP or ACD11 when exciting at 370 nm using AV-C1P and 3-perylenoyl-PC. **D)** Excitation and emission profiles for Me4-BODIPY-C1P (green) and C18-DiI (orange). **E)** FRET signal changes induced by CPTP or ACD11 when exciting at 485 nm and using Me4-BODIPY-C1P and C18-DiI. **F)** Illustration showing real-time emission data obtained using the FRET approach for measuring CPTP and GLTP lipid transfer specificity when monitoring AV-lipid emission at 415 nm. **G)** Schematic illustrating *in vitro* measurement of SL intermembrane transfer via FRET loss.

Figure S2

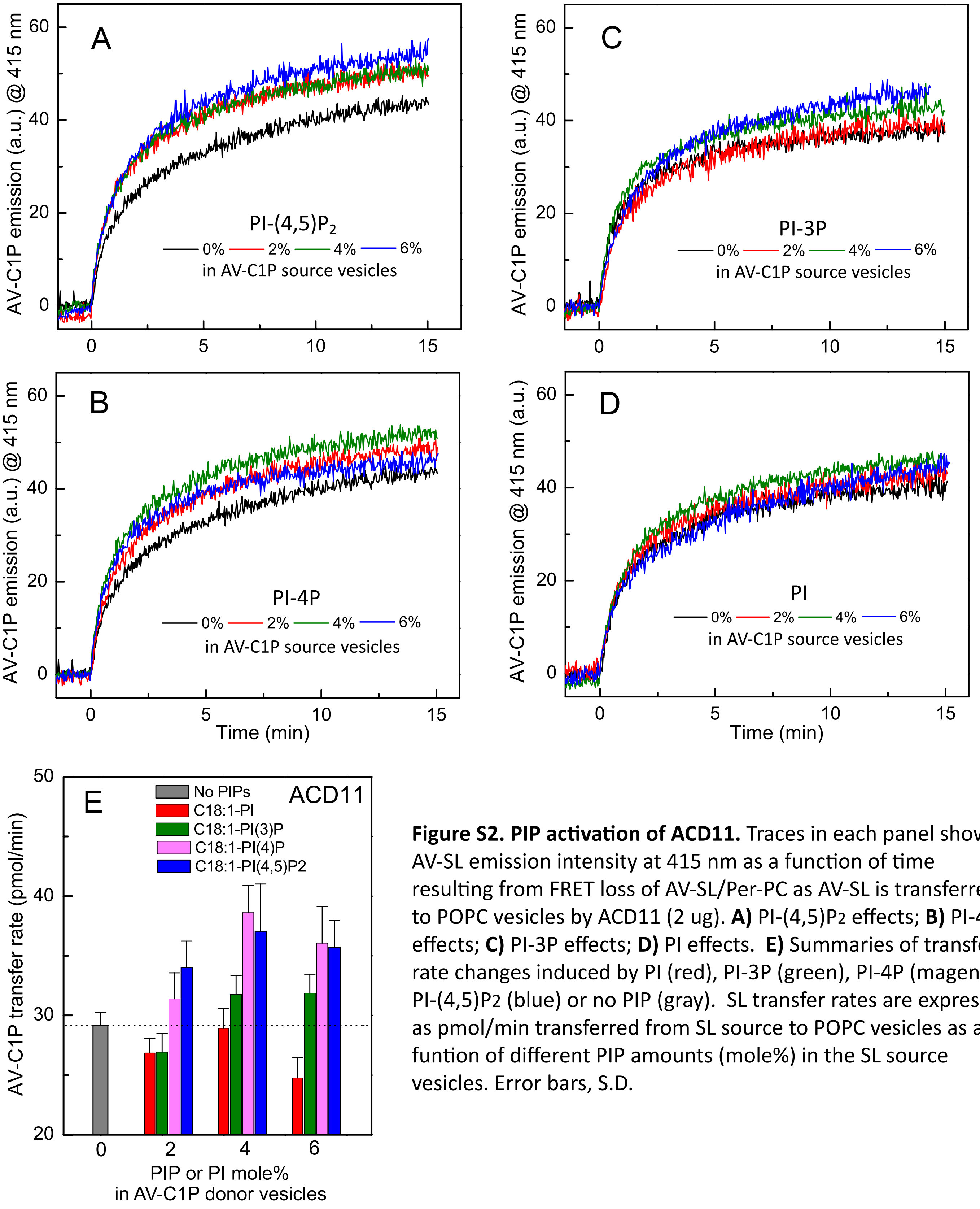

Figure S2. PIP activation of ACD11. Traces in each panel show AV-SL emission intensity at 415 nm as a function of time resulting from FRET loss of AV-SL/Per-PC as AV-SL is transferred to POPC vesicles by ACD11 (2 ug). **A)** PI-(4,5)P_2 effects; **B)** PI-4P effects; **C)** PI-3P effects; **D)** PI effects. **E)** Summaries of transfer rate changes induced by PI (red), PI-3P (green), PI-4P (magenta) PI-(4,5)P_2 (blue) or no PIP (gray). SL transfer rates are expressed as pmol/min transferred from SL source to POPC vesicles as a funtion of different PIP amounts (mole%) in the SL source vesicles. Error bars, S.D.

Figure S3

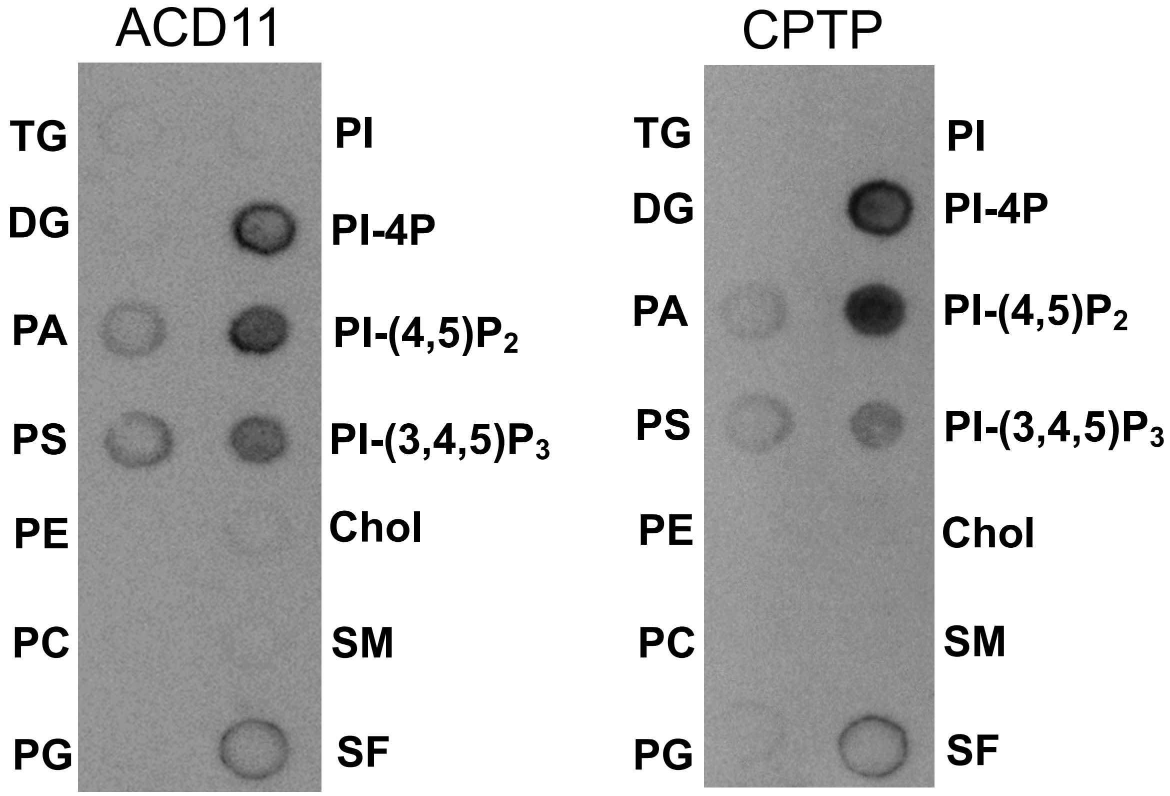

Protein-Lipid Overlay. **<u>Lipid Key</u>:** TG = triglyceride; DG = diglyceride; PA = phosphatidic acid; PS = phosphatidylserine; PE = phosphatidylethanolamine; PC = phosphatidylcholine; PG = phosphatidylglycerol; PI = phosphatidylinositol; PI4P = PI-4phosphate; PI-(4,5)P_2 = PI-(4,5) bisphosphate = PIP2; PIP-(3,4,5)P_3 = PI-(4,5) triphosphate = PIP3; Chol = cholesterol; SM = sphingomyelin; SF = sulfatide

Figure S4

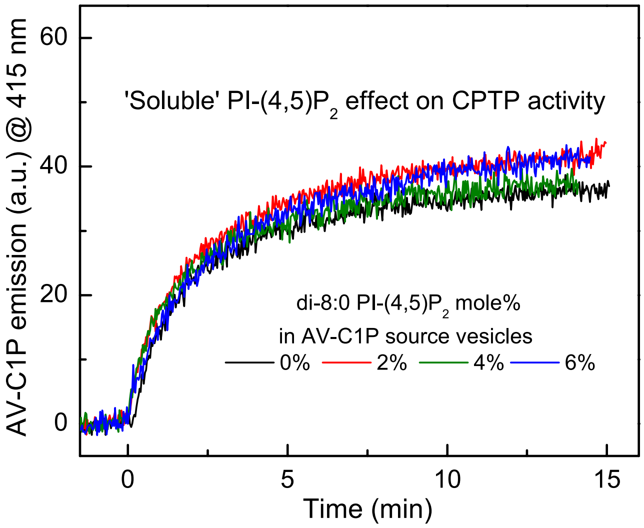

Figure S4. 'Soluble' PI-(4,5)P_2 fails to activate CPTP. Traces represent AV-SL emission intensity measured at 415 nm as a function of time resulting from the loss of AV-SL/Per-PC FRET as AV-SL is transferred to POPC vesicles by CPTP (2 μg).

Figure S5

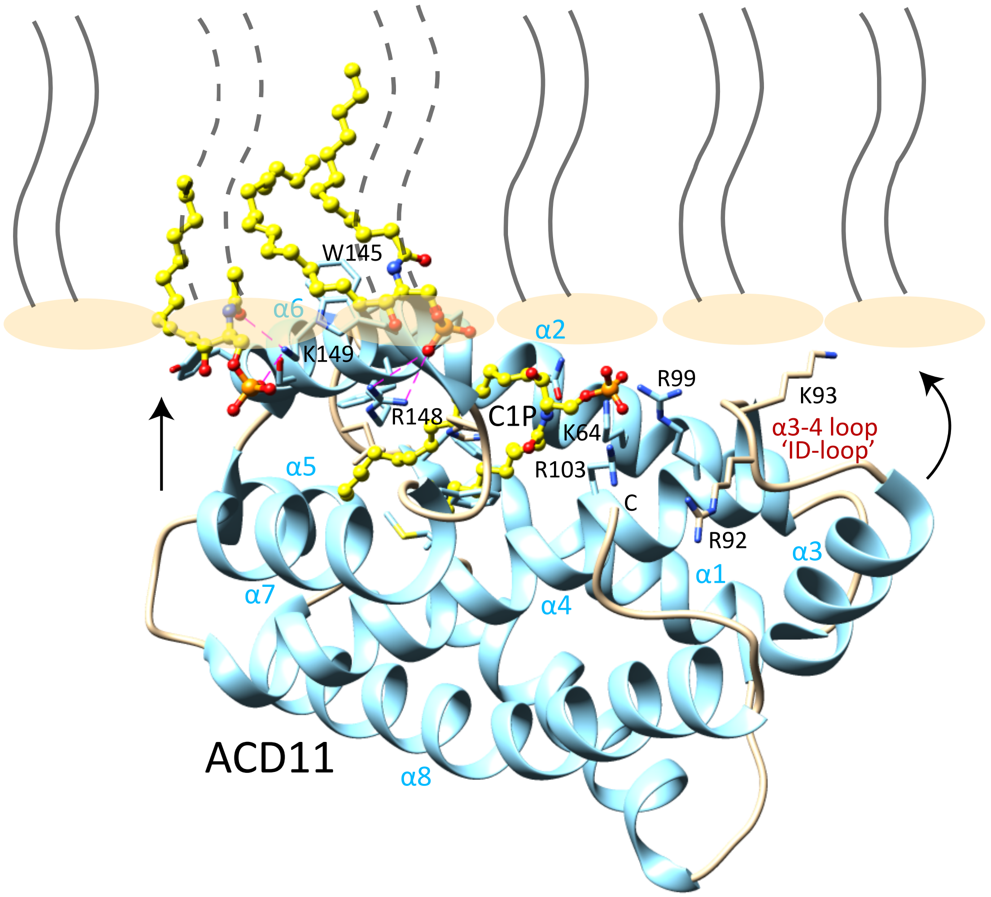

Figure S5. OPM-based model for ACD11 interaction with phosphoglyceride bilayers. OPM modeling for ACD11 complexed with 12:0 C1P (PDB: 4nti; 2.90 Å resolution) reveals favorable positioning of the α3-α4 helices connecting loop ('ID-loop') and the α6-helix and their respective R92/K93 and R148/K149 cationic motifs for acting as potential interaction sites for PIP headgroup binding.

Figure S6

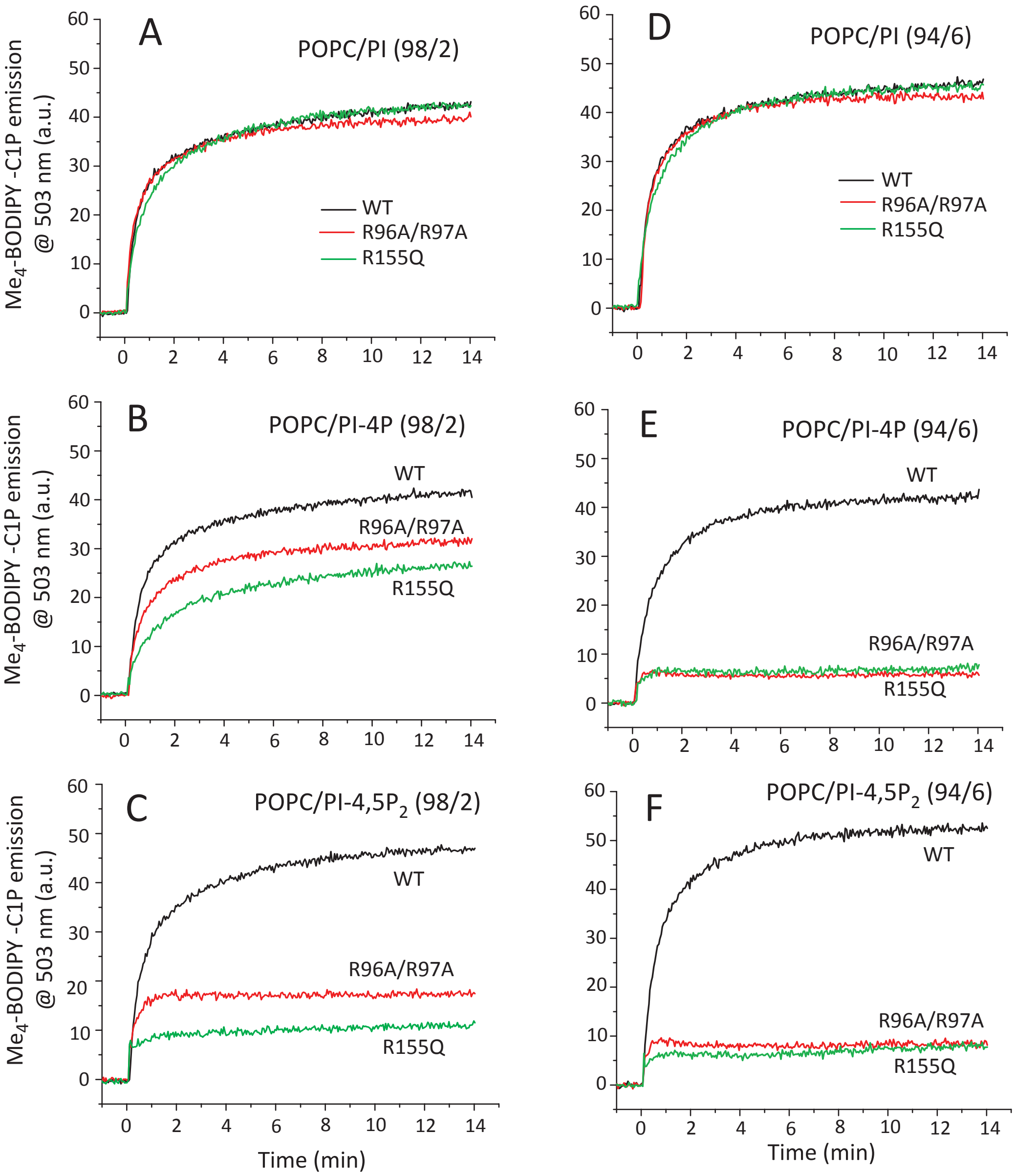

Figure S6. Mutation mapping identifying di-Arg motifs as PIP sites affecting CPTP-mediated C1P transfer. Traces in each panel show BODIPY-SL emission intensity measured at 503 nm as a function of time resulting from FRET loss of BODIPY-SL/C18-DiI when BODIPY-SL is transferred to POPC vesicles. SL donor vesicle matrix compositions are: **A)** POPC/PI (98:2); **B)** POPC/PI-4P (98:2); **C)** POPC/PI-(4,5)P_2 (98:2); **D)** POPC/PI (94:6); **E)** POPC/PI-4P (94:6); and **F)** POPC/PI-(4,5)P_2 (94:6). In each panel, wtCPTP (black trace), CPTP-R96A/R97A (red trace) and CPTP-R155Q (blue trace) are compared

Figure S7

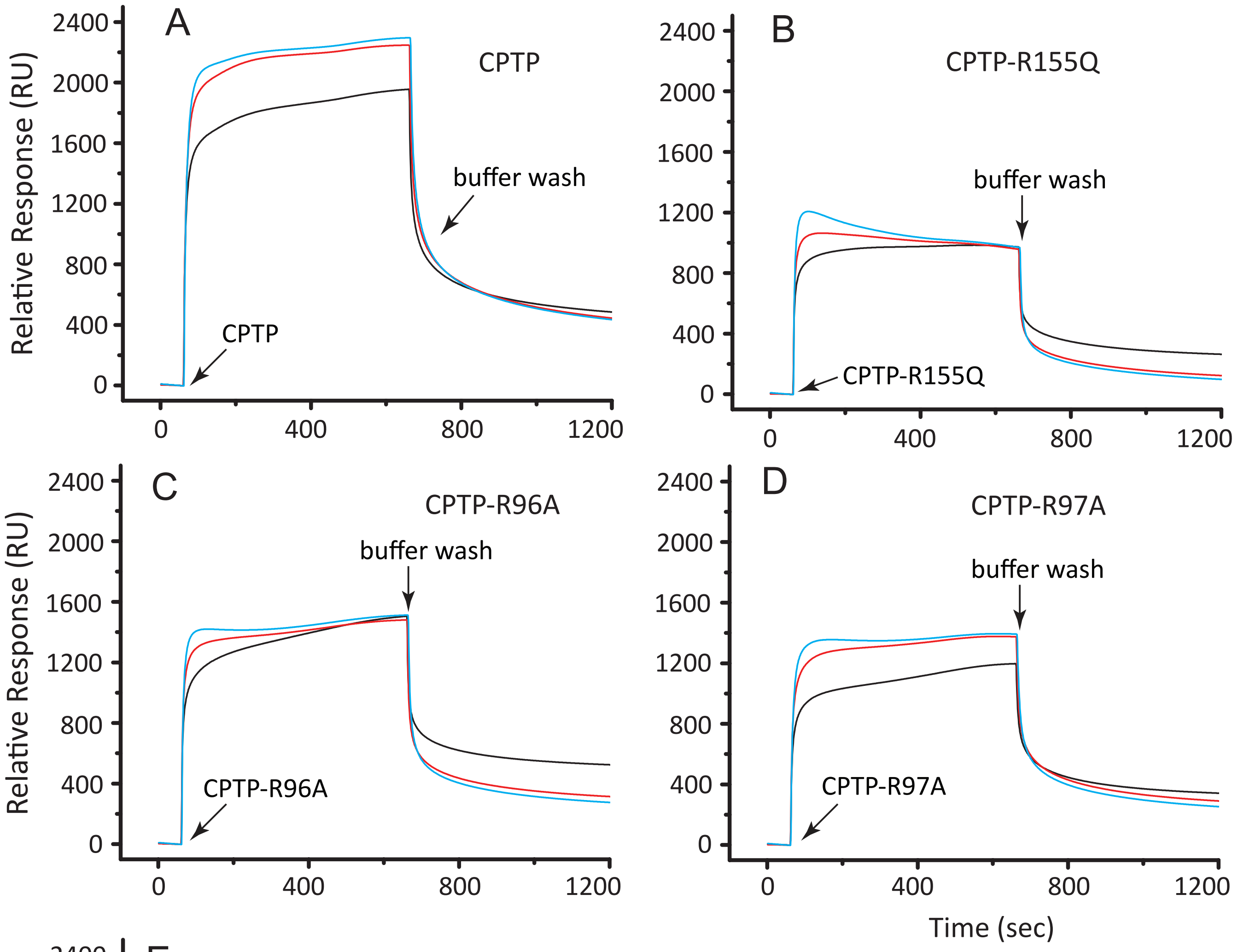

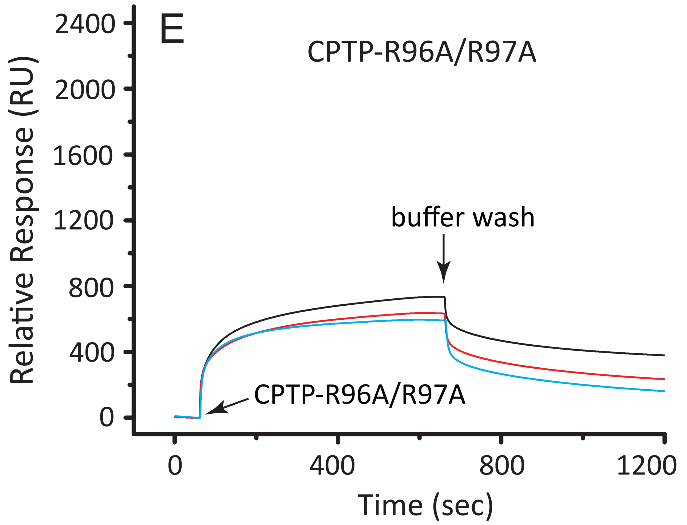

Figure S7. CPTP partitioning to POPC vesicles containing PI-(4,5)P_2 but not C1P. SPR analyses of CPTP mutatn adsorption and desorption to/from immobilized membrane vesicles of differing lipid composition. **A)** wtCPTP; **B)** CPTP-R155Q; **C)** CPTP-R96A; **D)** CPTP-R97A; **E)** CPTP-R96A/R97A. Injections are indicated by the 1st arrows and switches to buffer wash, by the 2nd arrow. In all panels, PI-(4,5)P_2 amounts of 0 (balck traces), 5 mole% (red traces) and 10 mole% (cyan traces) are shown for vesicles adsorbed to the L1 Sensor Chip (for more details, see Exptl. Proc.)

Figure S8

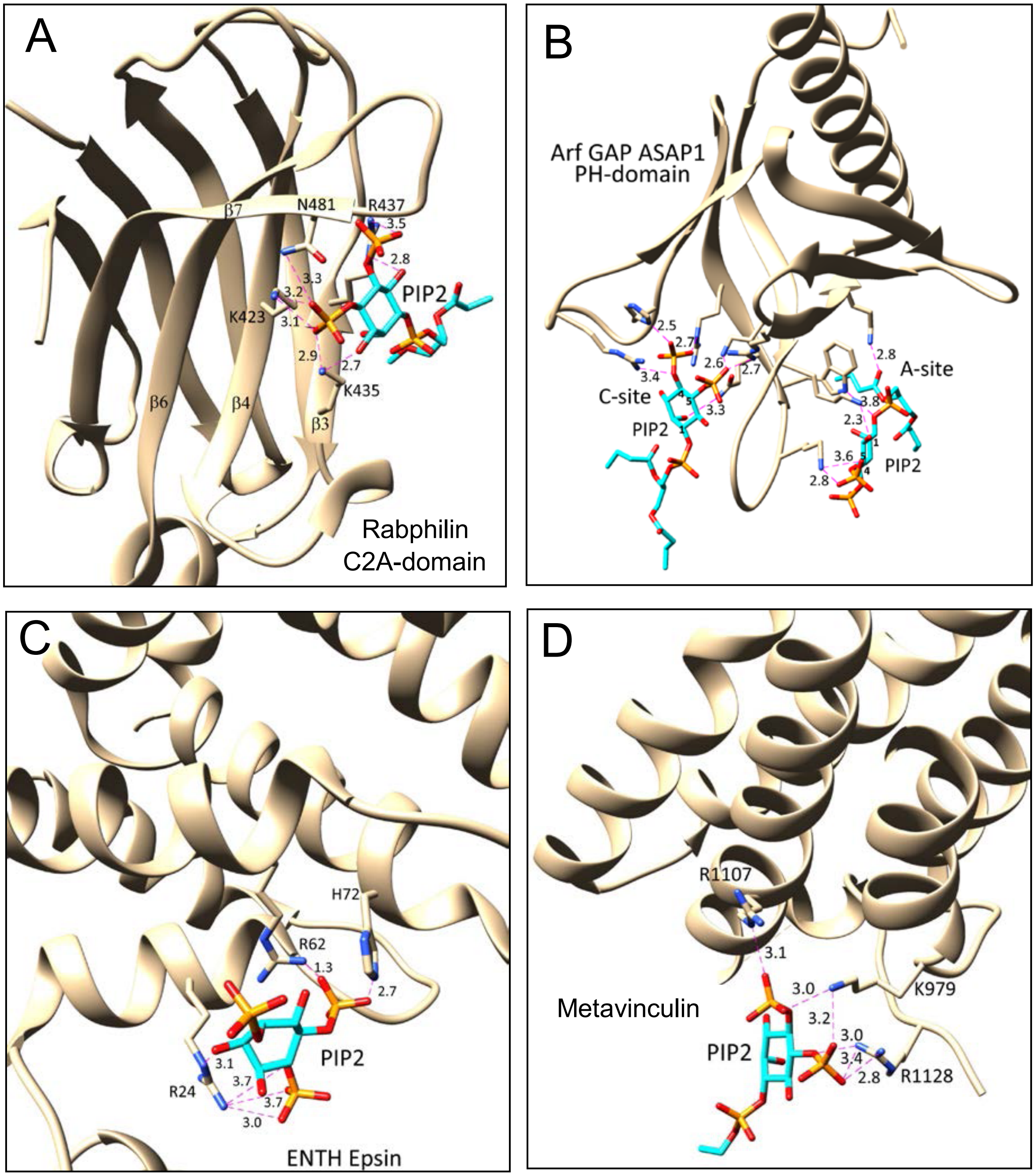

Figure S8. PI-(4,5)P_2 binding motifs in other peripheral amphitropic membrane proteins. **A)** Rabphilin C2A-domain (PDB: 4NSO); **B)** Arf GAP ASAP1 PH-domain (PDB: 5C79), C-site = canonical site & A-site = atypical site for PIP2; **C)** ENTH domain (PDB: 5NO7); **D)** Metavinculin (PDB: 5LOD). Ribbon representation is used with oxygen (red), nitrogen (blue), phosphate (orange) and PI-(4,5)P_2 carbon (cyan).

Figure S9

A

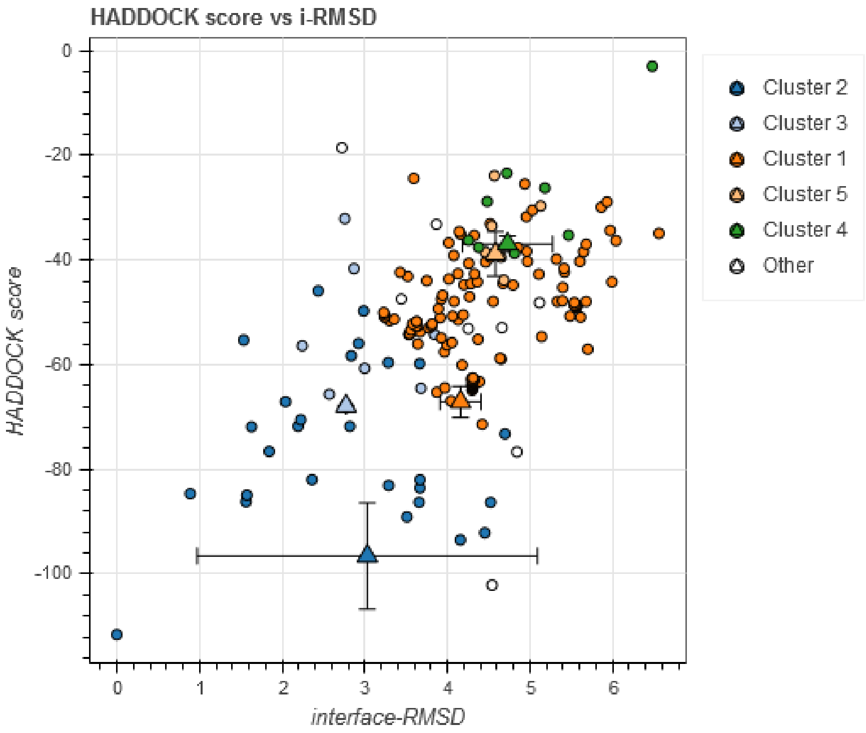

B

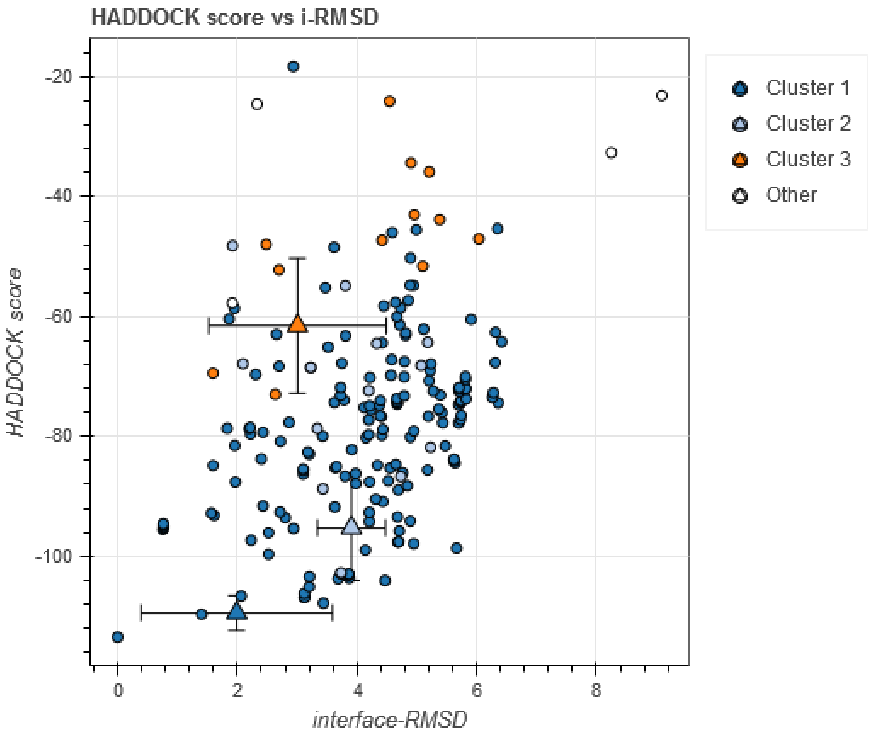

Figure S9: Scatter plots of HADDOCK results. HADDOCK scores versus interface RMSD for CPTP/PI-(4,5)P_2 complexes using R96/R97 **(A)** or R155/R156 **(B)** as ambiguous interaction restraints (AIRs).
